# When the Second Language Takes the Lead: Neurocognitive Processing Changes in the First Language of Adult Attriters

**DOI:** 10.3389/fpsyg.2017.00389

**Published:** 2017-03-30

**Authors:** Kristina Kasparian, Karsten Steinhauer

**Affiliations:** ^1^Neurocognition of Language Laboratory, School of Communication Sciences and Disorders, McGill UniversityMontreal, QC, Canada; ^2^Centre for Research on Brain, Language and MusicMontreal, QC, Canada

**Keywords:** neuroplasticity, first language attrition, second language acquisition, event-related potentials, language processing, crosslinguistic influence, relative clauses, language exposure

## Abstract

Although research on multilingualism has revealed continued neuroplasticity for language-learning beyond what was previously expected, it remains controversial whether and to what extent a second language (L2) acquired in adulthood may induce changes in the neurocognitive processing of a first language (L1). First language (L1) attrition in adulthood offers new insight on neuroplasticity and the factors that modulate neurocognitive responses to language. To date, investigations of the neurocognitive correlates of L1 attrition and of factors influencing these mechanisms are still scarce. Moreover, most event-related-potential (ERP) studies of second language processing have focused on L1 influence on the L2, while cross-linguistic influence in the reverse direction has been underexplored. Using ERPs, we examined the real-time processing of Italian relative-clauses in 24 Italian-English adult migrants with predominant use of English since immigration and reporting attrition of their native-Italian (Attriters), compared to 30 non-attriting monolinguals in Italy (Controls). Our results showed that Attriters differed from Controls in their acceptability judgment ratings and ERP responses when relative clause constructions were ungrammatical in English, though grammatical in Italian. Controls’ ERP responses to unpreferred sentence constructions were consistent with garden path effects typically observed in the literature for these complex sentences. In contrast, due to L2-English influence, Attriters were less sensitive to semantic cues than to word-order preferences, and processed permissible Italian sentences as outright morphosyntactic violations. Key factors modulating processing differences within Attriters were the degree of maintained L1 exposure, length of residence in the L2 environment and L2 proficiency – with higher levels of L2 immersion and proficiency associated with increased L2 influence on the L1. To our knowledge, this is the first demonstration that high levels of L2 proficiency and exposure may render a grammatical sentence in one’s native language ungrammatical. These group differences strongly point to distinct processing strategies and provide evidence that even a “stabilized” L1 grammar is subject to change after a prolonged period of L2 immersion and reduced L1 use, especially in linguistic areas promoting cross-linguistic influence.

## Introduction

### First Language (L1) Attrition

First language (L1) attrition allows us to study the impact of the second language (L2) on the native-language in a context of prolonged L2 immersion and reduced L1 use, usually after immigration to a new country (Köpke and Schmid, 2004). A number of behavioral studies have shown that attrition is typically detectable in the domain of lexical-semantics (de Bot, 1996; Köpke, 1999; Hulsen, 2000; Paradis, 2003, 2007; Köpke and Schmid, 2004; Montrul, 2008; Opitz, 2011), whereas findings have been mixed in the domain of morphosyntax (Ammerlaan, 1996; Gürel, 2004; Tsimpli et al., 2004; Kim et al., 2010; Schmid, 2010; Schmid and Köpke, 2011; Sorace, 2011). Moreover, it has been shown that L1 attrition is far less pervasive in adults than in children (see reviews by Köpke, 2004 and Köpke and Schmid, 2004), in whom L1 linguistic patterns are argued to be deeply entrenched (Tokowicz and MacWhinney, 2005) and stabilized (Bylund, 2009).

While some behavioral studies have provided evidence of L2 influence on the grammar of adult L1 attriters (see Schmid, 2011), neurocognitive investigations of L1 attrition are still scarce (Pallier, 2007; Datta, 2010; Schmid, 2013; Kasparian, 2015). A recent event-related-potential (ERP) study by Bergmann et al. (2015) tested German–English attriters’ processing of gender agreement violations and verb form combinations, compared to monolingual native speakers of German. Both groups showed the same ERP response (a posterior P600 effect) when processing gender agreement violations. However, when processing verb form violations, only the attriters showed an additional N400 effect prior to the posterior P600, suggestive of potential influence from their L2-English grammar, in which verb form violations have been found to elicit such biphasic N400+P600 responses (Sabourin and Stowe, 2008). The authors did not report whether these response patterns were modulated by any factors related to the attriters’ bilingual experience, such as L1 proficiency, exposure/use, length of residence (LoR), etc. Attriters scored lower than native-monolinguals on a written proficiency measure (German *C*-test, Schmid and Dusseldorp, 2010) but did not differ in their acceptability ratings nor on an offline gender assignment task. The authors concluded that the predominantly used L2 engenders little change to the processing of the deeply entrenched L1 grammar, and that ERPs are less susceptible to attrition effects than active language production.

The opposite was found in a recent ERP study of number agreement processing in Italian by Kasparian et al. (2016). Although attriters (L1-Italian, L2-English) scored numerically lower on a number of written and oral proficiency measures, the only behavioral difference from native-controls that reached significance was the attriters’ longer response times during the online acceptability judgment task. In contrast, L1 processing routines examined at two target points within a sentence revealed both qualitative and quantitative ERP differences between groups. Subject-verb number mismatches elicited a robust N400 effect in attriters but not in native-controls, reflecting attriters’ stronger expectations for agreement between a sentence-initial NP and the verb, likely as a result of English word-order influence. Attriters also differed from native-monolinguals in the sentence-repair processes indexed by the late posterior P600 (Hagoort and Brown, 2000; Carreiras et al., 2004; Molinaro et al., 2008). Interestingly, the late P600 was larger (i.e., more similar to native-monolinguals) in attriters with more frequent L1-Italian use.

As the experimental sentences in Kasparian, Vespignani, and Steinhauer tested combinations of both local- and non-local number agreement mismatches between three inflected constituents (noun, verb, and modifier), it seems likely that more complex morphosyntactic manipulations resulted in greater processing differences between attriters and native-monolinguals, compared to Bergmann et al. (2015). The present study aims to more directly examine L1 changes induced by the L2 grammar by testing the real-time processing of complex linguistic structures that operate differently in Italian and English, namely relative clause constructions.

### Relative Clause Processing

The comprehension of relative clauses has been studied extensively across languages, with both offline and online measures. These studies have generally demonstrated that subject relative clauses (e.g., *The reporter that attacked the senator admitted the error*) are easier to process than object relative clauses (e.g., *The reporter that the senator attacked admitted the error*) in most languages (Schriefers et al., 1995; De Vincenzi, 1996; Hagoort and Brown, 2000; Friederici et al., 2001; Traxler et al., 2005; but see Carreiras et al., 2010 for an opposite preference in Basque). In the comprehension of temporarily ambiguous subject-first and object-first sentences, the initial tendency is to disambiguate the sentence toward a subject-first reading (Clifton and Frazier, 1989; De Vincenzi, 1991; Schriefers et al., 1995; Bader and Meng, 1999; Schlesewsky et al., 2000). A mismatch between the preferred/expected structure that is automatically computed online and the actual input leads to longer reading times and poorer accuracy in the less preferred condition. Several theories have been proposed to explain such processing preferences, ranging from syntactic accounts (e.g., Clifton and Frazier, 1989), working memory (WM) load (e.g., Frazier and Fodor, 1978), the simultaneous influence of syntactic and non-syntactic information (e.g., MacDonald et al., 1994), usage frequency (e.g., MacDonald et al., 1994; McRae et al., 1998), to universal complexity (e.g., MacWhinney, 1982).

A number of ERP studies have shown that unpreferred relative clause sentences create garden-path effects and require revision once the disambiguating element (e.g., number of the verb) is encountered. These processes have been associated with a centro-parietal P600 effect and/or a preceding early frontal positivity, depending on the processing difficulty involved in constructing the sentence interpretation (Mecklinger et al., 1995; Steinhauer et al., 1997; Friederici et al., 2001).

The centro-parietal P600 is an effect that has not only been elicited by outright syntactic violations (e.g., Neville et al., 1991; Hagoort et al., 1993; Friederici et al., 1996, 1999), but also by violations of structural preference in garden-path sentences (Osterhout and Holcomb, 1992, 1993; Osterhout et al., 1994), as well as in response to less expected syntactic structures (Kaan et al., 2000). In these studies, the P600 effect has sometimes been discussed as reflecting processes of diagnosis and re-analysis or repair that are required to arrive at a well-formed sentence (see ‘*Diagnosis and Repair’* theory by Fodor and Inoue, 1998, discussed in Friederici et al., 2001 for garden-path sentences). Larger and more prolonged P600s between 500 and 900 ms typically reflect costlier repair processes (Hagoort and Brown, 2000; Carreiras et al., 2004; Silva-Pereyra and Carreiras, 2007; Molinaro et al., 2008). The P600 response has also been associated with a mismatch between expected and actual semantic (thematic) roles assigned to NP arguments by the critical verb (Kuperberg et al., 2003; see also Hoeks et al., 2004; Kim and Osterhout, 2005). According to this view, a processing cost is incurred when semantic biases are overridden by the semantic relationships dictated by the syntactic structure of the sentence (see also Kolk et al., 2003; van Herten et al., 2005). In line with this interpretation, sentence revision and repair has been shown to be more difficult when both NPs are animate (Mak et al., 2002, 2006; Traxler et al., 2002). An ERP study of object relative clauses (Weckerly and Kutas, 1999) reported a P600 effect on both the relative clause verb and the matrix verb when thematic roles based on animacy were contradicted by the thematic roles actually assigned by the verb, that is for sentences where the inanimate (rather than the animate) noun was the subject of the verb (e.g., *The novelist that the movie inspired praised the director…*).

A somewhat earlier posterior positivity has also been discussed as a P300 component (Mecklinger et al., 1995; Friederici and Mecklinger, 1996; Steinhauer et al., 1997; Friederici et al., 2001). The P300 (specifically the P3b) has been described as reflecting a process of WM updating that may be triggered by having encountered an unexpected syntactic structure. Studies investigating garden-path effects in German object relative-clauses (Mecklinger et al., 1995; Steinhauer et al., 1997) revealed a positivity around 350 ms for participants with a high reading span – an effect that was taken to reflect a revision process that is less cognitively demanding (Friederici and Mecklinger, 1996) than revision processes which trigger a late and longer-lasting posterior P600 (see also Hagoort et al., 1999; Hagoort and Brown, 2000).

A more frontally distributed positivity (often termed “frontal P600”) has also been reported for non-preferred sentence continuations or complex ambiguous sentences (Osterhout and Holcomb, 1992; Hagoort et al., 1999; Van Berkum et al., 1999; Friederici et al., 2002; Kaan and Swaab, 2003; Penolazzi et al., 2005). Similar frontal positivities have also been discussed as belonging to the P300 family (specifically a P3a; cf. Bowden et al., 2013) and reflecting surprise (Squires et al., 1975; Polich, 2007) or an attentional shift when processing an unexpected stimulus (Näätänen and Galliard, 1983).

The P600 is often accompanied by preceding negativities between 300 and 500 ms, although this pattern is more typical for morphosyntactic violations than for garden-path sentences (e.g., Friederici, 2002; Molinaro et al., 2011). In reading studies, such negativities are most often left-lateralized [i.e., the left-anterior negativity (LAN); see also Steinhauer et al., 2010 for left-temporal negativities (LTN)] and reflect mismatches with structure-based expectations (Molinaro et al., 2011). While LANs and LTNs are typically viewed as the most likely ERP response preceding the morpho-syntactic P600, negativities may have a broader distribution near midline and are interpreted as N400 components, reflecting either additional lexical processing costs (eADM model: Bornkessel and Schlesewsky, 2006; Brouwer et al., 2012) or also mismatches with structure-based morphological expectations (Tanner et al., 2013). Most interpretations of the N400 in sentence contexts are linked to lexical processing difficulties, during either word retrieval or semantic integration (Kutas and Federmeier, 2011). Both P600 and N400 amplitudes show a gradual increase the stronger the linguistic anomaly and the more difficult the underlying processes.

### Cross-Linguistic Differences in Italian and English Relative Clauses

Cross-linguistic differences in morphosyntactic properties and in semantic biases make the study of relative clause comprehension relevant for bilingual speakers, particularly when the two linguistic systems operate differently in sentence processing preferences, as is the case for English and Italian.

The two languages have been shown to differ in the cues that speakers make use of during sentence interpretation. As English has a strict word-order and a less detailed system of morphological markers, English speakers rely heavily on word-order for sentence interpretation. Conversely, Italian has a relatively free word-order and rich morphological marking system, thus number agreement and semantic information (e.g., animacy, thematic roles) are more salient cues than word-order in identifying the subject of a sentence (see “*Competition Model*”; Bates et al., 1982; MacWhinney, 1987; MacWhinney and Bates, 1989; see also Bornkessel-Schlesewsky and Schlesewsky, 2009; Bornkessel-Schlesewsky et al., 2011).

In terms of word-order, Italian relative clauses have been described as having four syntactically acceptable constructions (i.e., two different word-orders that are *both* compatible with subject- and object-first relative clauses; see **Table 1**). Sentences may follow a NP-[V-NP] structure (henceforth “V-NP”) or a NP-[NP-V] structure (henceforth “NP-V”) in which the relative pronoun “*che*” (= that/who) is directly followed by the second NP rather than by the verb. Although all four constructions are syntactically acceptable, NP-V-subject constructions have been described as having a low usage frequency, as they occur in poetry or songs, but less frequency in everyday Italian (Di Domenico and Di Matteo, 2009; as confirmed by acceptability ratings from native Italian speakers that we collected prior to creating our final stimuli). Given these four potential sentence constructions, the pronoun “*che*” can refer either to the subject or object of the relative clause; thus, the disambiguation of the sentence relies on semantic information and/or number agreement with the verb (Penolazzi et al., 2005; Di Domenico and Di Matteo, 2009).

**Table 1:** An example of experimental sentences is provided for each condition.

Condition	
(1) V-NP-subject	Il poliziotto (S) che arresta i ladri (O) registra i nomi. *The policeman (S) that arrests the thieves (O) registers the names.*
(2) V-NP-object^∗^	I ladri (O) che arresta il poliziotto (S) attendono in macchina. *The thieves (O) that arrests the policeman (S) wait in the car*.
(3) NP-V-subject^∗^	Il poliziotto (S) che i ladri (O) arresta registra i nomi. *The policeman (S) that the thieves (O) arrests registers the names*.
(4) NP-V-object	I ladri (O) che il poliziotto (S) arresta attendono in macchina. *The thieves (O) that the policeman (S) arrests wait in the car*.

*English translations are presented in italics. The target noun is underlined and subject/object roles are indicated in parentheses. The asterisk (^∗^) marks those conditions which are morphosyntactic violations in English*.

In a behavioral reading study of Italian native-speakers, Di Domenico and Di Matteo (2009) tested the acceptability and processing difficulty – as reflected by reading times – of these word-orders, using reversible sentences with animate nouns, where verb number was the only disambiguating cue (e.g., *The director that criticized_-sing_ the workers anticipated the holidays*). Results showed that the V-NP-subject construction was the most preferred^1^ and was associated with the fastest reading times on the verb of the relative clause. Increased reading times registered for the V-NP-object and NP-V-subject conditions were taken as evidence of revision and integration processes, after a preferred sentence structure was initially pursued. The authors argued in favor of two processing phases: a first phase where an automatically developed sentence structure is revised, followed by a second phase further downstream where the revised interpretation and assigned thematic and syntactic roles are confirmed. Once these processes have taken place, no further reading delays were incurred on subsequent words.

In contrast, English only allows for V-NP-subject and NP-V object word-orders, whereas VP-NP-object and NP-V-subject sentences are outright syntactic violations (**Table 1**), regardless of whether the sentence interpretation is supported by semantic/thematic information and/or number agreement. It was therefore of interest to examine whether the processing routines underlying Italian relative clause comprehension may have changed as a result of prolonged daily exposure to English.

### Cross-Linguistic Influence in Sentence Processing in Bilinguals

It has been widely attested that a bilingual’s two languages are simultaneously active during the real-time processing of only one language. Evidence of influence of the L1 during online L2 morphosyntactic processing has been demonstrated in eye-tracking (Frenck-Mestre and Pynte, 1997) and ERP studies (e.g., Sabourin, 2003; Ojima et al., 2005; Tokowicz and MacWhinney, 2005; Kasparian et al., 2010; Foucart and Frenck-Mestre, 2011, 2012; White et al., 2012). Research has also examined the factors at play in modulating the degree of L1–L2 influence – linguistic similarity, L2 proficiency and exposure levels have been shown to affect the extent of L1-transfer and the degree of native-like-ness in the L2 (see reviews by Kotz, 2009 and Caffarra et al., 2015). Modulations of cross-linguistic transfer, in both lexical-semantic and morphosyntactic domains, have been explained in terms of relative frequency of use and activation thresholds, with the more dominant language (generally the L1) associated with a higher baseline activation level and a better efficiency in regulating cross-linguistic competition (e.g., McDonald, 1987; MacWhinney, 1992; Kroll and Stewart, 1994; Jared and Kroll, 2001; Dijkstra and Van Heuven, 2002; Gollan et al., 2008).

In contrast, studies that have explored transfer in the *reverse* direction (L2 onto L1) have been more limited, particularly in morphosyntax (Frenck-Mestre and Pynte, 2000; Linck et al., 2009; Whitford and Titone, 2012; Timmer et al., 2014). An eye-tracking study by Dussias and Sagarra (2007) tested attachment preferences in temporarily ambiguous relative clauses (e.g., *the brother_1_ of the actress_2_ that_?_ went to Boston*) in Spanish–English bilinguals with either limited or extensive L2 immersion experience, compared to native-Spanish monolingual speakers. Differences in relative clause attachment preferences were found between groups; while monolingual Spanish speakers and bilinguals with limited immersion experience reliably preferred to attach the relative clause to the first NP as Spanish speakers do (e.g., Cuetos and Mitchell, 1988; Carreiras and Clifton, 1993; Carreiras et al., 2004; Mitchell and Cuetos, 1991, Unpublished), bilinguals with extensive L2-English exposure attached the relative clause to the second NP as English speakers do (Frazier and Clifton, 1996; Carreiras and Clifton, 1999; Dussias, 2001, 2003). Interestingly, the differences between the bilingual groups held when L2 proficiency was matched. The authors take these results to support the permeability of the L1 system as a result of extensive L2 exposure. These findings can be explained within the same theoretical frameworks outlined above, only that the L2 has become the predominantly used language, rather than the L1.

It can be argued that attriters belong on the same language experience continuum as those L2 learners who have been extensively immersed in the L2, whether or not attrition consists of a more extreme shift from L1 to L2.

### The Present Study

Using ERPs, the present study examined the real-time processing of four different word-orders of Italian relative clauses in a group of Italian-English adult migrants who have been predominantly exposed to English since immigration and who have unanimously reported experiencing attrition in Italian (Attriters), compared to 30 non-attriting native-speakers in Italy (Controls). In one of the earliest ERP studies of adult L1 attrition and the first to systematically manipulate a complex aspect of morphosyntax to yield a paradigm where the L1 and L2 either converge or diverge, our main aim was to determine whether there were quantitative and/or qualitative differences in L1 processing patterns in attriters, due to L2 immersion. Secondly, we studied whether L1 processing was modulated by factors such as L2-influence, L1/L2 proficiency, L1/L2 use or LoR in the L2 environment. Finally, to address the open question of whether attrition effects are more pervasive in online comprehension or in behavioral/production tasks, we compared online and offline responses.

We expected the groups to differ most on the two critical conditions [(2) V-NP-o and (3) NP-V-s], as those sentences are syntactically acceptable in Italian but not in English. If native-Controls and Attriters were to process these sentences as permissible but unpreferred (due to the mismatch with syntactic/semantic preferences and/or lower usage frequency^2^), then they may show an increased reliance on semantic cues (N400 effect for unpreferred conditions; cf. Mecklinger et al., 1995) and engage in a revision process similar to what has been documented for garden-path sentences (frontal positivity and/or P600). Instead, if Attriters were to show influence from L2-English morphosyntax, we would expect them to process V-NP-o and NP-V-s sentences as morphosyntactic violations, eliciting ERP responses that differ in latency and scalp topography from those elicited by the native-Controls. According to most authors (e.g., Molinaro et al., 2011), attriters would therefore elicit ERP responses associated with the early detection (LAN) and diagnosis/repair (robust P600) of a violation, and not show evidence of relying on semantic cues for disambiguation. Since some authors have reported that a subset of subjects elicit N400s for morpho-syntactic violations (e.g., Tanner and Van Hell, 2014), finding an N400 in Attriters would be somewhat ambiguous. In terms of individual differences, we would expect that the Attriters who differ most from Controls in their L1-processing are those individuals with higher L2 proficiency, higher L2 exposure and/or a longer LoR. Such findings would show a shift from L1-cues to L2-cues with increased L2 proficiency and exposure in adult attriters (see McDonald, 1987).

It is worth noting that our experimental design tests attriters’ processing of sentences that are syntactically *correct* in their L1 (but not in their L2), rather than the typical approach of testing their responses to L1 morphosyntactic violations. Thus, while the common finding is that less exposed or less proficient speakers (usually L2 learners) elicit smaller or delayed ERP effects than native-speakers or more proficient L2 learners, in the case of the present study, we would expect the reverse, namely that L1 attriters would elicit *stronger* morphosyntactic violation effects, as a result of predominant English (L2) exposure.

## Materials and Methods

### Participants

Twenty-four Italian native-speakers (14 female; *M* age: 36; Range: 25–50) who had relocated to Canada in adulthood [*M* age at immigration (AoA of English^3^): 28.2 years; Range: 18–40; *M* length of residence: 11 years; Range: 1–26] were tested at McGill University in Montreal, Canada. Attriters reported limited exposure/use of their L1-Italian (*M* daily L1 exposure: 14.92%; Range: 1–40%), and described changes or difficulties as a result of their predominant L2-English use (*M* daily L2 exposure: 69.54%; Range: 60–96%). Thirty Italian native-speakers were tested as a control group at the University of Trento in Rovereto, Italy (17 female; *M* age: 31; Range: 25–54). They had little to no exposure to second languages (including English and Italian dialects), which we operationally defined as less than 5 h per week. All participants except one were right-handed and with no known history of neurological disorders.

### Background Measures

A background questionnaire collected participants’ demographic (age, gender, and education) and language information. Attriters answered additional questions about their immigration history, context and amount (in hours per week and % per day) of L1/L2 exposure and use, motivation for L1 maintenance and L2 mastery, and identity/attitudes toward each language and culture. Both groups completed four proficiency measures : (1) A written self-report measure where they rated their L1 proficiency level on a scale from 1 to 7 in listening, reading, pronunciation, fluency, vocabulary, and grammatical ability; (2) A written *C*-test (Italian version: Kraš, 2008), where they were asked to fill in the blanks in 5 short texts in which 20 words in each text had been partially deleted; (3) A written error-detection test (Kasparian, 2015), where they had to detect and correct errors in two texts; and (4) A timed verbal semantic fluency task where they were asked to produce as many vocabulary items a given semantic category within 1 min. They also completed (1) a timed reading fluency task where they silently read and answered as many true-false statements as possible in 3 min (adapted into Italian based on Woodcock et al., 2003), and (2) the letter-number-sequencing task from the Italian WAIS-IV as a measure of WM (Orsini and Pezzuti, 2013). The purpose of these tasks was to ensure that any group differences were not a result of reading speed and/or WM differences. Group means are provided in **Table 2**. Attriters scored numerically lower on all four proficiency measures, though they did not differ significantly from Controls (*p* > 0.1).

**Table 2:** Group means (standard deviation) for Italian proficiency and control tasks (*p*s > 0.1).

Background measures	Controls (*n* = 30)	Attriters (*n* = 24)
Self-report of proficiency (7-point scale)	7 (0)	6.87 (0.2)
Listening comprehension	7 (0)	7 (0)
Reading comprehension	7 (0)	7 (0)
Pronunciation	7 (0)	6.96 (0.2)
Fluency	7 (0)	6.79 (0.6)
Vocabulary	7 (0)	6.63 (0.7)
Grammar	7 (0)	6.83 (0.4)
*C*-test (%)	96.3 (4.4)	95.2 (4.6)
Error-detection test (%)	90.0 (5.1)	89.5 (5.9)
Verbal semantic fluency (average of two categories)	23.4 (5.5)	21.5 (3.9)
Reading fluency (correct in 3 min)	71.6 (13.0)	75.3 (15.0)
Working memory		
Correct	11.2 (2.7)	11.9 (2.6)
Span	5.4 (1.1)	5.7 (1.1)

### Stimuli

Examples of each of the four experimental conditions are provided in **Table 1**. Each sentence began with a noun phrase (definite article + noun) which was either the subject or object of the verb in the relative clause, depending on the condition. The stimuli were based on the work of Di Domenico and Di Matteo (2009) in Italian and Mecklinger et al. (1995) in German. Noun-verb-noun triplets were created to form strong agent-patient relationships to disambiguate the sentence (e.g., attorney/convict/lawyer). Only animate nouns were used and psych verbs (fear, threaten, appreciate, love, etc.) were avoided, as they assign different theta roles (Bourguignon et al., 2012). There were no repetitions among nouns and verbs. Number was counterbalanced within each condition, such that half the sentences in each condition began with a singular subject noun, and half with a plural subject noun. Lemma frequency information for all nouns and verbs was obtained (CoLFIS database; Bertinetto et al., 2005). Both lemma frequency and length of NP1 (*M* freq.: 187.79; *M* length: 7.71) and NP2 (*M* freq.: 195.19; *M* length: 7.76) were matched across triplets (*p*s > 0.1). Sentences were nine words long; the target verb was either in fourth position (conditions 1 and 2) or sixth position (conditions 3 and 4). The final three words in the sentence were always the matrix-clause verb, a function word and a noun.

A set of 108 different sentences were constructed and realized in each of the eight conditions (four main conditions × singular/plural). Eight experimental lists were created such that, across lists, each sentence contributed equally to each condition, while no sentence was repeated within any of the experimental lists. Each participant also saw 216 filler sentences, which were part of the larger study (testing number agreement and lexical-semantic processing) and will be reported in separated papers (Kasparian and Steinhauer, 2016; Kasparian et al., 2016). Out of the total of 324 pseudorandomized stimuli (108 experimental and 216 fillers) per participant, 146 sentences (approximately 45%) were acceptable (grammatically and semantically), while 178 were expected to receive a rating of 3 or lower on a five-point rating scale (approximately 55%). Our stimuli were verified by two Italian native-speakers.

### Procedure

All participants provided written informed consent prior to their participation in the study. After completing the questionnaires and behavioral tasks, participants were fitted with the EEG cap and seated in a dimly-lit, sound-attenuated booth, at approximately 80 cm from the computer monitor with a *Cedrus* seven-button RB-740 response box placed in front of them (Cedrus Corporation, San Pedro, CA, USA). Participants were instructed that their task would be to rate the acceptability of various Italian sentences on a scale from 1 (unacceptable) to 5 (perfect). We used a rating scale rather than a binary acceptability judgment task in order to better capture the range of permissibility of the relative clause constructions, which were not outright violations in Italian. Moreover, among native-speakers, a rating scale may be more sensitive to subtle group differences than yes/no decisions. Words were presented in white 40-point Arial font characters, at the center of a black background. Each trial began with the presentation of a white fixation cross for 500 ms, followed for 200 ms by a blank screen (ISI). Each word then appeared one at a time for 300 ms (+200 ms ISI). A visual prompt (“???”) followed the offset of the sentence-final word and remained on the screen until participants’ button press, after which an image of the blue eye appeared at the center of the screen for a 2000 ms interval for participants to blink their eyes. The next trial began after the blinking interval. Each session lasted approximately 3 h, including setup, short breaks and cap removal. All consent forms, materials and procedures were approved by the Ethics Review Board of each institution.

### EEG Recording and Analysis

The EEG was recorded continuously from 25 Ag/AgCl electrodes, 19 of which were electrodes mounted on a standard electro-cap according to the 10–20 system (Jasper, 1958), and six of which were external electrodes: four electro-oculogram (EOG) channels placed above and below the left eye (EOGV), and at the outer canthus of each eye (EOGH), as well as two reference electrodes placed on the mastoids (A1 and A2). All electrodes were referenced online to the left mastoid (A1). Impedances were kept strictly below 5 kΩ for scalp and reference electrodes, and below 10 kΩ for EOG electrodes. Signals were amplified using NeuroScan (Canada) and BrainVision (Italy) and filtered online with a band-pass filter of 0.1 to 100 Hz a sampling rate of 500 Hz. Data pre-processing and analyses were carried out using EEProbe (ANT, Enschede, Netherlands). Offline, EEG recordings were filtered with a phase-true 0.3–40 Hz band-pass filter. Trials containing artifacts due to blinks, eye-movements, and excessive muscle activity were rejected prior to averaging, using a moving-window (400 ms) standard deviation of 30 μV. On average, participants contributed 25 artifact-free trials per condition out of 27 trials, with no differences across conditions (*p*s > 0.1). One Attriter was excluded from the analysis due to exceedingly noisy trials.

Event-related potentials were analyzed separately for the V-NP and NP-V word-orders^4^ and were time-locked to the onset of the verb in the relative clause. For the V-NP contrast, the baseline correction was from -200 to 0 ms. For NP-V conditions, the baseline was set at 0 to 1200 ms, due to early differences in Attriters that created a baseline problem^5^. ERPs were quantified in time-windows corresponding to each component of interest, based on visual inspection of the data. For V-NP analyses, the time-windows were: (1) 300–400 (LAN/N400); (2) 650–850 (P600); (3) 850–1050 (late P600). For NP-V analyses: (1) 300–400 (LAN/N400); (2) 550–650 (frontal positivity); (3) 650–900 (P600); and (4) 900–1050 (late P600).

Repeated-measures ANOVAs were performed separately for 4 midline electrodes (Fz, Cz, Pz, and Oz) and 12 lateral electrodes (F3/4, C3/4, P3/4 and F7/8, T3/4, T5/6). Global ANOVAs for the midline sites included within-subject factors *Condition* (C: subject, object) and *Ant-Post* (AP: anterior, central, parietal, occipital). Lateral ANOVAs additionally included factors *Hemisphere* (left, right) and *Laterality* (lateral, medial). *Group* (G: Controls, Attriters) was the between-subjects factor. Greenhouse-Geisser correction was applied to analyses with more than two levels (e.g., *AP*). In these cases, the corrected *p*-values but original degrees of freedom are reported. Reported analyses are restricted to the midline only, except in cases where the lateral ANOVAs revealed additional effects. *Post hoc* analyses when following up multi-level main effects or interactions in ANOVAs were not affected by *post hoc* Bonferroni corrections; all significant *post hoc* analyses remained significant after correction for multiple comparisons (cf., Keppel and Wickens, 2004). Correlations were performed between all relevant participant factors (LoR, exposure, Italian proficiency, English proficiency) and experimental data (acceptability judgments, ERP effects quantified at a representative electrode in representative time-windows). In ANOVAs and correlational analyses, we do not report non-significant results unless motivated in specific contrasts, i.e., to emphasize the absence of an effect in one group or one condition compared to another.

## Results

### Acceptability Judgments

**FIGURE 1:**
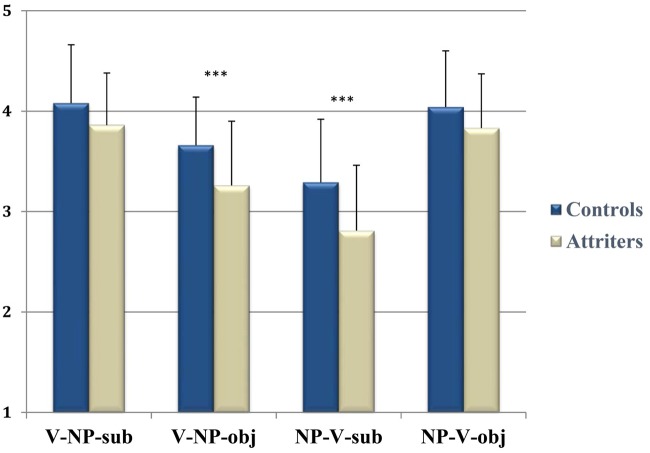
**Group acceptability ratings on a scale from 1 (completely unacceptable) to 5 (perfect) by condition**. Attriters rated V-NP-o and NP-V-s sentences significantly less favorably than Controls (^∗∗∗^*p* < 0.005). Error bars represent standard deviation.

Acceptability ratings (1–5) for each sentence condition are shown in **Figure 1**. Overall, the acceptability rating results were in line with the findings from Di Domenico and Di Matteo (2009) where the order of acceptability was condition # 1 < 4 < 2 < 3 in Italian native-speakers. The repeated-measures ANOVA with factor *Condition* (C: 1, 2, 3, 4) and *Group* (G: Controls, Attriters) revealed a significant C main effect [*F*(3,153) = 104.184, *p* < 0.0001] and a *C* × *G* interaction [*F*(3,153) = 2.60, *p* < 0.05]. Follow-up analyses of the *C* main effect showed that, across both groups, conditions 1 and 4 were significantly more accepted than conditions 2 and 3 (*p* < 0.0001 for all corresponding pairwise comparisons). Moreover, *Condition* 2 was rated as more acceptable than *Condition* 3 (*p* < 0.0001), whereas the two grammatical conditions 1 and 4 only differed numerically from each other (*p* = 0.4). Most importantly, the *C* × *G* interaction indicated that (compared to Controls) Attriters were more likely to reject those sentences that are ungrammatical in English. That is, Attriters (provided significantly lower ratings than Controls for V-NP-o [*F*(1,52) = 10.40, *p* < 0.005] and NP-V-s [*F*(1,52) = 8.434, *p* < 0.005] conditions, while not differing on V-NP-s and NP-V-o (*p*s > 0.1). As expected, Attriters judged the two conditions that are outright grammatical violations in English as less acceptable in Italian than native-controls, suggesting influence from their L2-English grammar. In addition, higher levels of Italian-L1 exposure were significantly correlated with more positive acceptability ratings for these unpreferred conditions (V-NP-o: *r* = 0.367, *p* < 0.005; NP-V-s: *r* = 0.318, *p* < 0.01).

In line with the interpretation that Attriters treated the two critical conditions as outright morphosyntactic violations, we found that Attriters’ acceptability ratings for the two unpreferred relative-clause conditions were not found to differ statistically from ratings the same participants provided in the same experimental session for outright morphosyntactic number agreement violations (*p*s > 0.1, see Kasparian et al., 2016). Conversely, the same native-Controls provided significantly higher acceptability ratings for the RC word-orders than for number agreement violations presented in the same experimental session, indicating that they did indeed consider these RC word-orders as more grammatically acceptable than the Attriters.

To better understand group differences and variability in ratings for the two critical conditions (V-NP-o and NP-V-s), participants were clustered into high-raters and low-raters by median split across all participants’ ratings^6^ (**Table 3**). For V-NP word-orders, a *Condition* (subject vs. object) main effect [*F*(1,53) = 51.28, *p* < 0.0001] was qualified by a significant interaction between *Condition* × *Rater Type* × *Group* [*F*(1,53) = 5.913, *p* < 0.05]. The *Group* (Controls vs. Attriters) × *Rater-Type* (High vs. Low) interaction was significant for V-NP-o sentences [*F*(1,53) = 7.49, *p* < 0.01], as “low-rater” Attriters rated the unpreferred V-NP-o condition significantly less favorably than even “low-rater” Controls. The trend followed the same direction for NP-V-s sentences, where we found a significant interaction between *Condition* × *Rater Type* [*F*(1,53) = 12.57, *p* < 0.01]. “Low-rater” Attriters rated the unpreferred NP-V-s sentences less favorably than “low-rater” Controls, although this numerical difference did not reach statistical significance (*p* = 0.7). There were no significant differences between the two “high-rater” subgroups of Controls vs. Attriters for either condition (*p*s > 0.1). The differences between the “low rater” subgroups suggest that there is more at play than individual variability among native Italian speakers.

**Table 3:** Mean (standard deviation) acceptability ratings per condition by group and rater-type.

	V-NP-s	V-NP-o	NP-V-s	NP-V-o
Low-rater Controls (*n* = 10)	3.61 (0.49)	3.27 (0.19)	2.64 (0.43)	3.64 (0.50)
High-rater Controls (*n* = 20)	4.31 (0.30)	3.86 (0.27)	3.67 (0.31)	4.28 (0.41)
Low-rater Attriters (*n* = 16)	3.71 (0.39)	2.96 (0.28)	2.51 (0.34)	3.65 (0.40)
High-rater Attriters (*n* = 7)	4.21 (0.39)	3.98 (2.58)	3.53 (0.33)	4.28 (0.26)

### Reaction Times

**FIGURE 2:**
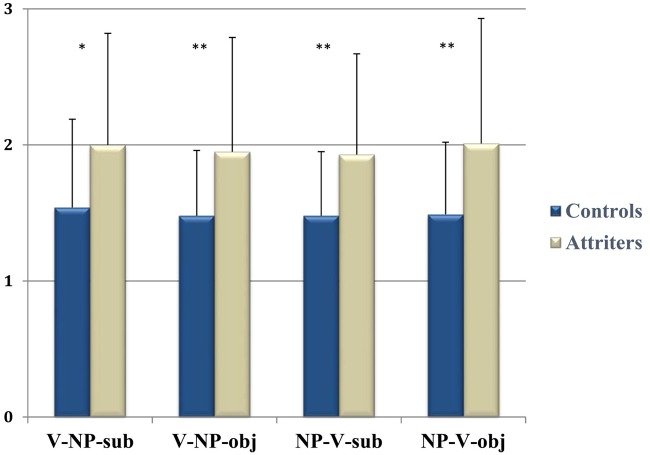
**Group reaction times (in seconds) by condition**. Attriters were consistently slower than Controls ^∗^*p* < 0.05, ^∗∗^*p* < 0.01. Error bars represent standard deviation.

Reaction times between the onset of the prompt and participants’ button-press are shown in **Figure 2**. The repeated-measures ANOVA with factors *Condition* (1, 2, 3, 4) and *Group* (G: Controls, Attriters) revealed a main effect of *Group* [*F*(1,52) = 7.547, *p* < 0.008], reflecting Attriters’ overall slower response times than Controls^7^. Contrary to previous results that unpreferred (object) and uncanonical (NP-V) sentences take longer to process (as in Di Domenico and Di Matteo, 2009), differences between conditions did not reach significance (*p*s > 0.1). This may be a result of our task (i.e., acceptability rating rather than a comprehension question) or the offline nature of this measure, as participants’ responses were given at the end of the sentence rather than on the target word, as is standard practice to avoid motor artifacts in the EEG.

### ERP Results for V-NP-Object vs. V-NP-Subject

**FIGURE 3:**
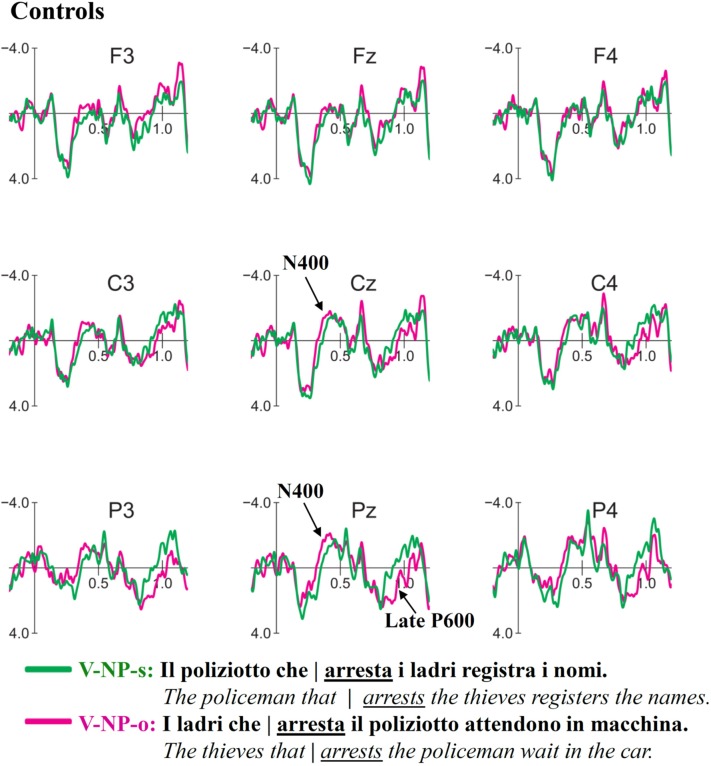
**Event-related-potentials (ERPs) elicited by the verb in response to V-NP-object sentences (pink) compared to V-NP-subject sentences (green) in Controls**. Time ranges depicted on the *x*-axis are relative to the onset of the verb of the relative clause (0 ms). Negative values are plotted up. Controls show an N400 effect followed by a late, posterior P600 effect.

**FIGURE 4:**
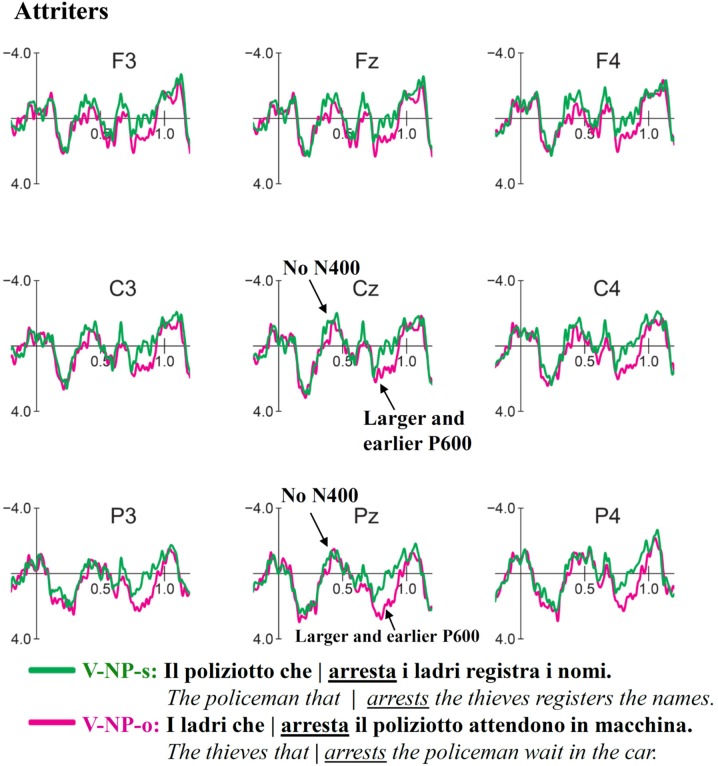
**Event-related-potentials elicited by the verb of the relative clause in V-NP-object sentences (pink) compared to V-NP-subject sentences (green) in Attriters**. Unlike Controls, Attriters do not elicit an N400 effect. The P600 effect is earlier, larger and broadly distributed in Attriters.

**FIGURE 5:**
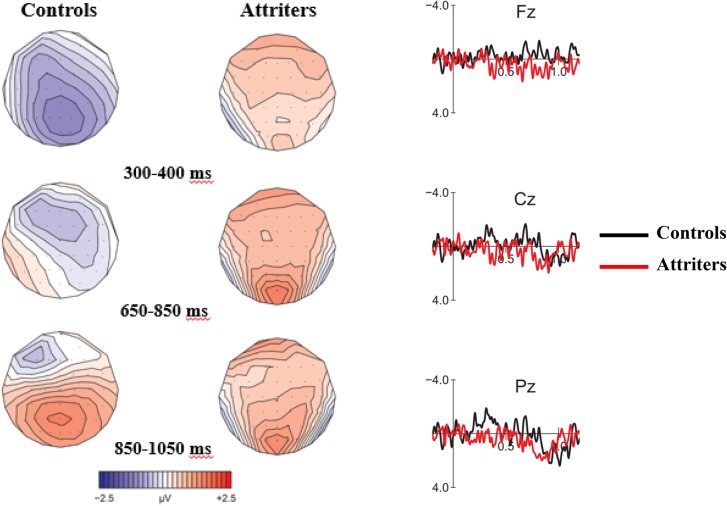
**Voltage maps (left) and ERP difference waves (right) illustrating condition differences (V-NP-object minus V-NP-subject) in Controls and Attriters for each time-window of interest**.

Grand average ERP waveform for V-NP (Object vs. Subject) conditions time-locked to the verb of the relative clause are presented in **Figure 3** (Controls) and **Figure 4** (Attriters). In Controls, unpreferred (though syntactically acceptable) object relative clauses elicited a broadly distributed N400-like negativity (300–400 ms) and late posterior P600 (850–1050 ms). In Attriters, there is no evidence of a negativity, and the P600 effect appears to have an earlier onset, larger amplitude, and broader distribution (650–1050 ms). Group differences for relevant time intervals are illustrated with topographical maps in **Figure 5**.

#### N400 (300–400 ms)

The global midline ANOVA between 300 and 400 ms revealed a significant *C* × *G* interaction [*F*(1,52) = 7.56, *p* < 0.01], due to the presence of a negativity in Controls [*F*(1,29) = 9.78, *p* < 0.005] but not Attriters (*p*s > 0.1). No interactions with topographical factors pointing toward a left and/or anterior scalp distribution reached significance in the lateral ANOVA (*p*s > 0.1). The negativity in response to object-relative sentences was therefore consistent with a N400 effect.

**FIGURE 6:**
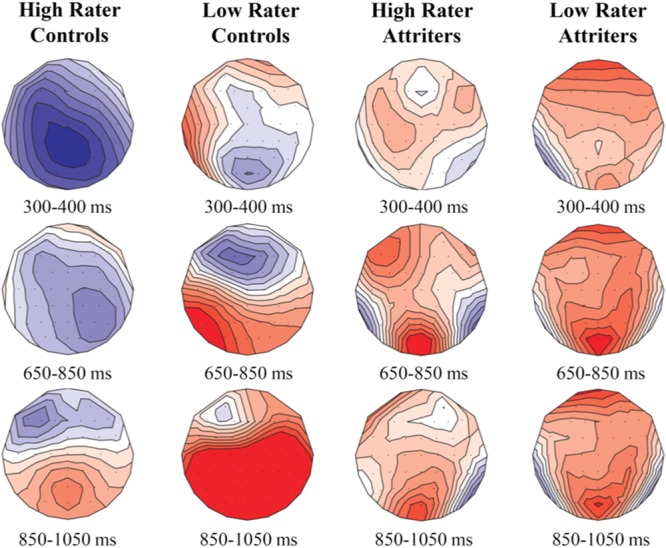
**Voltage maps illustrating condition differences (V-NP-object minus V-NP-subject) in subgroups of high and low acceptability raters in Controls and Attriters for each of the time-windows of interest**. High rater controls elicited a robust N400 compared to low rater Controls. Neither of the subgroups of Attriters elicited an N400 response.

To aid in the interpretation of the functional significance of the N400, we examined ERP patterns in relation to acceptability ratings. Our hypothesis of enhanced reliance on semantic cues in Controls is supported by the finding that Controls who provided higher acceptability ratings for V-NP-o sentences (high raters) elicited a significant N400 [*F*(1,19) = 12.96, *p* < 0.005], whereas low rater Controls elicited a weak N400 that was not statistically reliable (*p*s > 0.1)^8^. The N400 was therefore associated with higher acceptability rather than with a violation effect. In contrast, within Attriters, the N400 was absent not only in low but also high raters who did not even show a trend toward an N400 (*p*s > 0.8), suggesting an insensitivity to semantic cues in sentence interpretation^9^. These patterns are illustrated in **Figure 6**.

In line with our hypothesis that Attriters were influenced by L2-English grammar in which word-order prevails over semantic cues, correlations revealed less negative amplitudes for object-relatives at Pz in Attriters with a longer LoR (*r* = 0.346, *p* < 0.05) and with higher L2-English proficiency scores (*C*-test: *r* = 0.313, *p* = 0.07).

#### Early P600 (650–850 ms)

In the early time window for the P600, the midline ANOVA showed a significant *C* × *AP* [*F*(3,156) = 4.56, *p* < 0.05] and a marginal *C* × *G* interaction [*F*(1,52) = 3.62, *p* = 0.06]. Group follow-ups showed that Attriters elicited a broadly distributed P600 [*C*: *F*(1,23) = 5.03, *p* < 0.05] whereas *C* × *AP* was marginal [*F*(3,69) = 3.15, *p* = 0.06], but the P600 did not even approach significance in Controls (*p*s > 0.6). Attriters show an enhanced processing cost in this early P600 time-window when processing V-NP-o sentences, compared to native-Controls.

Within Attriters, a larger P600 amplitude at Pz was associated with a longer LoR (*r* = 0.346 *p* < 0.05) and higher L2-English proficiency scores (Semantic fluency: *r* = 0.347; *p* < 0.05), suggesting that increased L2 immersion and proficiency is associated with stronger morphosyntactic violation effects, as a result of L2 influence on the L1.

#### Late P600 (850–1050 ms)

At the midline, a significant main effect of *C* [*F*(1,52) = 6.71, *p* < 0.01] was qualified by a significant *C* × *AP* interaction [*F*(3,156) = 8.13, *p* < 0.0001], reflecting the posterior distribution of the late P600 [*F*(1,52) at Cz < Pz < Oz]. The 3-way *C* × *AP* × *G* interaction was marginal [*F*(3,156) = 2.26, *p* = 0.08] but reached significance in the lateral ANOVA [*F*(2,104) = 6.48, *p* < 0.01]. Group follow-ups revealed a significant *C* × *AP* interaction in Controls [midline: *F*(3,87) = 10.16, *p* < 0.0005; lateral: *F*(3,87) = 17.91, *p* < 0.0001] but not Attriters (*p*s > 0.1). In Attriters, only a main effect of *C* was marginally significant at midline sites [*F*(1,23) = 3.26, *p* = 0.08]. Thus, overall, Attriters elicited a weaker and more broadly distributed P600 than Controls in this later time-window.

#### Time-Window Analysis of P600

To investigate whether the groups differed significantly in the latency of the P600, we conducted an additional analysis including factor time-window (TW) comparing the two time-windows reported above (i.e., 650–850 vs. 850–1050 ms). The midline ANOVA revealed a *TW* × *C* × *G* interaction [*F*(1,52) = 9.01, *p* < 0.005], which was driven by a *TW* × *C* interaction in Controls [*F*(1,29) = 13.81, *p* < 0.005] but not Attriters (*p* > 0.1). The lateral ANOVA showed that the distribution also differed across groups and TWs [*TW* × *C* × *AP* × *G*: *F*(2,104) = 3.82, *p* < 0.05]. Group follow-ups revealed a significant *TW* × *C* × *AP* interaction in Controls [*F*(2,58) = 9.45, *p* < 0.005], whereas no interactions with factor TW reached significance in Attriters (*p*s > 0.1). This analysis further supported the finding that the P600 differed in latency and distribution between groups, with Attriters showing a more robust, earlier and more broadly distributed P600 effect for V-NP-o sentences than Controls.

### ERP Results for NP-V Subject vs. NP-V Object

**FIGURE 7:**
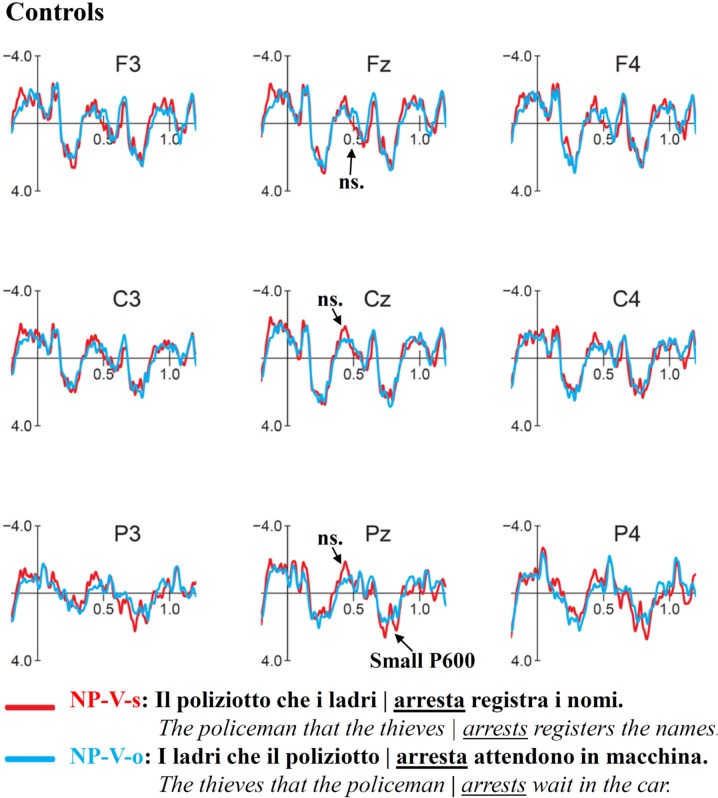
**Event-related-potentials elicited by the verb of the relative clause in NP-V-subject sentences (red) compared to NP-V-object sentences (blue)**. Controls only elicited a small posterior P600 effect in response to NP-V-subject sentences.

**FIGURE 8:**
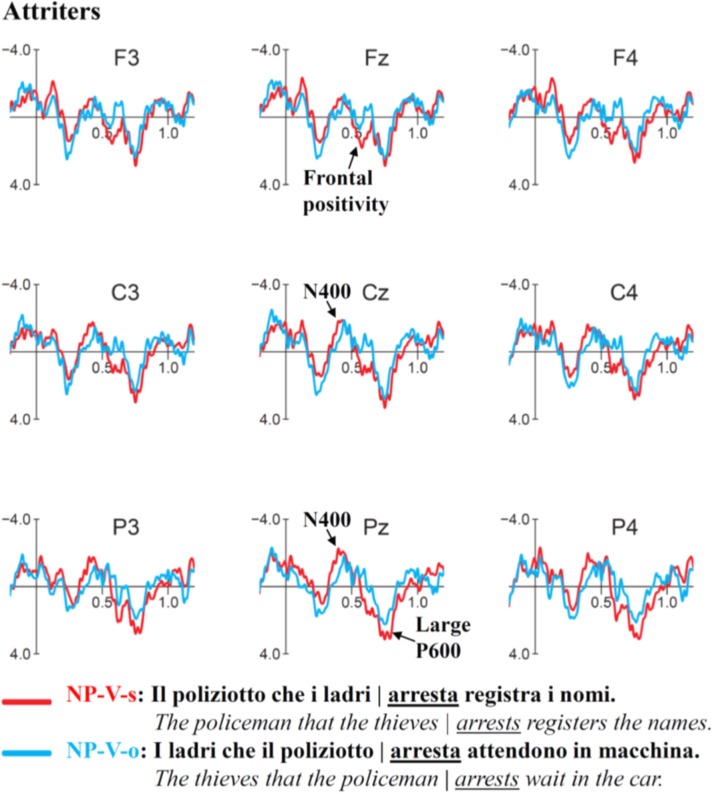
**Event-related-potentials elicited by the verb of the relative clause in NP-V-subject sentences (red) compared to NP-V-object sentences (blue)**. Attriters elicited an N400-like negativity, followed by an early fronto-central positivity and a large P600 effect for NP-V-subject sentences.

**FIGURE 9:**
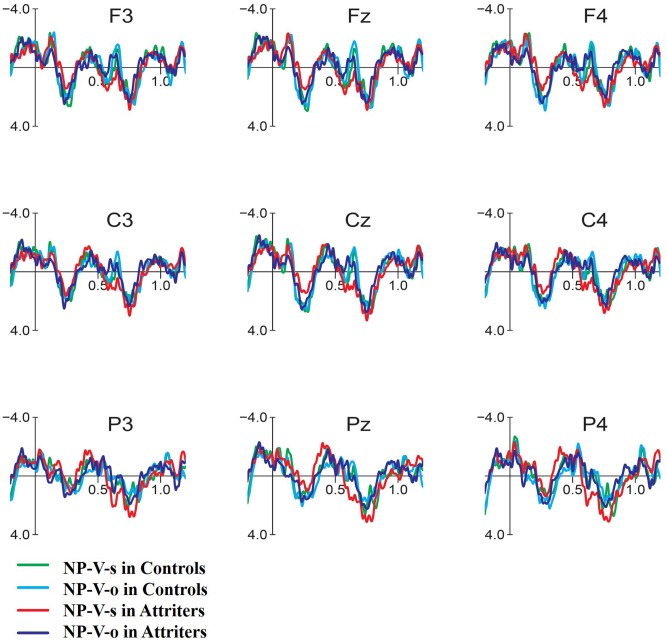
**Event-related-potentials elicited by the verb of the relative clause in each of NP-V conditions (subject and object) in Controls and Attriters**. The NP-V-subject condition in Attriters (red) is the condition that differs most from other conditions.

Grand average ERP waveforms for NP-V (Subject vs. Object) conditions time-locked to the verb of the relative clause are presented in **Figure 7**. In Controls (**Figure 7**), unpreferred (though syntactically acceptable) subject relative clauses with this word-order elicited only weak differences compared to the object relative clause: a small frontal positivity visible at Fz (550–650 ms) was followed by a small posterior P600 beginning around 700 ms. In contrast, Attriters (**Figure 8**) elicited a large negativity that extended to frontal sites (300–400 ms), a numerically larger fronto-central positivity (550–650 ms) and a larger, earlier and seemingly less posterior P600 effect than Controls. Comparing both conditions in each of the two groups indicates that the English violation condition (NP-V-s) in Attriters is the condition that stands out when all four ERP waves are plotted together (**Figure 9**).

#### N400 (300–400 ms)

The midline ANOVA revealed a significant main effect of *C* [*F*(1,51) = 6.12, *p* < 0.05] and a marginal *C* × *AP* interaction [*F*(3,153) = 2.87, *p* = 0.06]. The lateral ANOVA additionally showed a marginal *C* × *G* interaction [*F*(1,51) = 3.13, *p* = 0.08], which when followed-up demonstrated a negativity in Attriters [*F*(1,22) = 4.31, *p* < 0.05] but not Controls (*p* > 0.1).

#### Frontal Positivity (550–650 ms)

On the midline, a significant main effect of *C* [*F*(1,51) = 13.30, *p* < 0.001] was qualified by significant *C* × *AP* [*F*(3,153) = 5.86, *p* < 0.01] and *C* × *G* interactions [*F*(1,51) = 4.11, *p* < 0.05]. Follow-ups confirmed that the positivity in Attriters was frontal in distribution [*C* × *AP*: *F*(3,66) = 4.69, *p* < 0.05; Fz > Cz > Pz] and robust [*C*: *F*(1,22) = 11.51, *p* < 0.005] compared to Controls (*p*s > 0.1). Rater-type (low vs. high) did not modulate the frontal positivity, and the most relevant factor was Group.

#### P600 (650–900 ms)

The midline ANOVA yielded significant *C*[*F*(1,51) = 6.13, *p* < 0.05] and *C* × *AP* effects [*F*(3,153) = 4.26, *p* < 0.01], reflecting the prominence of the positivity at Pz. The interaction between *C* × *G* was also significant [*F*(1,51) = 4.56, *p* < 0.05], which when followed up revealed a *C* main effect in Attriters [*F*(1,22) = 7.95, *p* < 0.01] but not Controls (*p* > 0.1). The interaction between *C* × *AP* × *G* did not reach significance (*p* > 0.1). Note that Controls showed no indication of a parietal P600^10^.

#### Late P600 (900–1050 ms)

Unlike for V-NP conditions, the late P600 effect elicited in the NP-V subject condition was statistically shared by Controls and Attriters, as interactions with *G* did not reach significance (*p*s > 0.1). A significant *C* × *AP* interaction [*F*(3,153) = 3.79, *p* < 0.05] pointed to the posterior distribution of the positivity (Fz: *p* = 0.9; Cz: *p* = 0.3; Pz: *p* < 0.05; Oz: *p* < 0.05).

## Discussion

The present study examined the real-time L1 processing in adult Italian-migrants who had been predominantly exposed to English since immigration to Canada and who unanimously reported experiencing attrition in Italian, compared to non-attriting native-speakers still living in Italy. Our aim was to determine whether qualitative and/or quantitative differences would be found in the processing of complex relative clause constructions, due to cross-linguistic influence from the L2.

We expected Attriters to process Italian relative clause sentences whose structure would be ungrammatical in English (V-NP-object and NP-V-subject) as morphosyntactic violations, despite the presence of semantic cues to aid in the disambiguation of thematic roles. We were interested in whether such effects would also be present in Attriters’ behavioral performance, and whether ERP responses would be modulated by factors such as proficiency, exposure and LoR.

### Acceptability Ratings

Our main finding was that the critical conditions (V-NP-o and NP-V-s) were rated as outright morphosyntactic violations by Attriters but not by Controls. First, in native-monolinguals, the order of acceptability of the four word-order conditions [(i.e., 1, 4, 2, 3) in order of decreasing acceptability] was the same as in a previous study by Di Domenico and Di Matteo (2009), although the results of the two studies cannot be compared directly due to a difference in judgment scale and given that the stimuli in Di Domenico and Di Matteo were reversible sentences with two animate nouns, where verb number was the only disambiguating cue (e.g., *The director that criticized*_-sing_ *the workers anticipated the holidays*). Given that we introduced a semantic bias in our sentences to disambiguate the agent of the verb (e.g., policeman/arrest/thief), it may be that our sentences were more readily acceptable by native-Controls.

Attriters, contrary to Controls, provided significantly lower acceptability ratings for V-NP-o and NP-V-s sentences. Crucially, the groups did not differ in their acceptability judgments of V-NP-s and NP-V-o sentences which are syntactically acceptable in both Italian and English. Further evidence that Attriters treated V-NP-o and NP-V-s sentences as morphosyntactic violations comes from the finding that their ratings for these sentences did not differ statistically from ratings the same participants gave in response to Italian number agreement violations during the same experimental session. In contrast, Controls rated V-NP-o and NP-V-s sentences higher than the number agreement violations (see Kasparian et al., 2016). These results suggest cross-linguistic influence from English (L2) word-order during Italian (L1) sentence-reading. Given that acceptability judgments were provided at the end of each sentence and may not reflect online differences occurring at the critical sentence positions, it was of interest to determine whether and how these group differences would be reflected in real-time ERP responses.

### Processing of V-NP Sentences

Our ERP findings were in line with the acceptability judgment results and demonstrate that the rating differences between groups resulted from online processing differences at disambiguating target words. In response to V-NP-object Italian sentences which are unpreferred compared to subject relative clauses but still syntactically acceptable, Controls showed an N400 effect between 300 and 400 ms at the disambiguating verb, indicating that they were sensitive to the semantic cues that served as extra disambiguating information to identify the subject of the sentence (Penolazzi et al., 2005; Di Domenico and Di Matteo, 2009). This pattern is reminiscent of the findings of a German ERP study by Mecklinger et al. (1995), who also observed an enhanced N400 for non-preferred object relative structures, but only when semantic cues conflicted with initial parsing preferences, and only in their group of ‘fast comprehenders.’ German, like Italian (but unlike English), also has a free word-order, and disambiguation of relative clauses depends on verb inflection and semantic cues (see Bornkessel-Schlesewsky and Schlesewsky, 2013, for a discussion of cross-linguistic differences).

Our finding of a larger N400 with *higher* acceptability judgment ratings (numerically even within Controls) further supported the view that the elicitation of the N400 was associated with more favorable responses and therefore did not index a violation effect. The N400 is reduced in subject relative-clauses where the verb (e.g., *arrests*) is both semantically primed by its preceding noun (e.g., *policeman*) *and* represents an action compatible with this preceding noun’s assumed theta role as an actor/agent. Conversely, in sentences that begin with the object (e.g., *thieves*), the enhanced N400 reflects that – despite a likely semantic priming effect – the verb may still be less expected (Van Petten and Kutas, 1991; Federmeier and Kutas, 1999; Kuperberg, 2007), as the verb violates the thematic role that had been computed online based on the first noun.^11^ In line with this, the ERP study on Italian subject/object constructions by Penolazzi et al. (2005) did not find an N400 effect preceding the reported positivities (P300 and P600), possibly due to the reversibility of their thematic roles (e.g., grandfather/kiss/child). This absence of an N400 is entirely in line with Mecklinger et al.’s (1995) findings for their ‘neutral’ (i.e., reversible) condition that lacked semantic biases.

Following the N400 effect, Controls also showed a late, posterior P600 response to V-NP-o relative to V-NP-s sentences. The relatively long latency of the P600 (compared to Attriters, see below) may partly be due to an ongoing N400 (i.e., the two components may have canceled each other out; cf. Steinhauer and Drury, 2012). It may also be linked to the specific type of garden-path effect involved to repair the input. As described in Penolazzi et al. (2005), even when the reader detects an unpreferred construction at the verb in the V-NP-o condition and attempts to revise it, the last constituent (subject) is not yet available; the expectation for the incoming NP to be assigned the subject role and the maintenance of an only partially constructed sentence representation in WM could incur a cognitive load and incur in a delay of syntactic integration processes, resulting in a late P600.

Attriters qualitatively and quantitative differed from Controls in their ERP responses. First, V-NP-o sentences did not elicit an N400 effect, contrary to Controls. In this respect, Attriters differed from Controls as a group overall, as even high rater Attriters did not show an N400 effect. In addition, correlations revealed less negative amplitudes in Attriters with higher L2-English proficiency scores and a longer LoR. This finding is in line with the argument that the N400 in Controls did not reflect a violation effect, as Attriters with more L2-English immersion were significantly less likely to show a negativity in the N400 time-window. These results suggest that Attriters were not sensitive to semantic cues (i.e., non-reversible agent-patient roles) to guide thematic role assignment during online sentence processing. Instead they seemed influenced by their L2-English grammar in which word-order is the most salient cue for sentence interpretation (Bates et al., 1982; McDonald, 1987; MacWhinney and Bates, 1989; Bornkessel-Schlesewsky and Schlesewsky, 2013). A similar shift in processing preferences has been shown in L2-dominant speakers in previous work by Dussias and Sagarra (2007) for relative clause attachment. We argue that L2-English immersion (with infrequent exposure to the Attriter’s L1-Italian) leads to changes in expectations pursued during online language processing. That is, due to the influence of strict English word-order, Attriters likely have a stronger expectation for the first noun of the sentence to be the subject NP (possibly to be assigned the role of Agent) such that they rely more heavily on word-order than on semantic information, compared to native-Italian monolingual speakers (Molinaro et al., 2011). As a consequence, the violation on the verb would be processed primarily as a morpho-syntactic agreement violation, whereas the semantic-thematic mismatch may be less salient than in the Control group^12^.

In line with this interpretation, the second ERP difference between Attriters and Controls was that Attriters showed an earlier, stronger and more broadly distributed P600 effect for V-NP-object sentences, which we interpreted as a reflection of a stronger anomaly (and possibly higher processing costs) compared to Controls. The finding of larger P600 amplitudes in participants who gave lower acceptability ratings to V-NP-o sentences supported our interpretation of a stronger violation effect in Attriters overall.

The P600 has been interpreted in various ways, perhaps most prominently as an index of morpho-syntactic error diagnosis and structural sentence re-analysis/repair (e.g., Fodor and Inoue, 1998, discussed in Friederici et al., 2001), with larger amplitudes reflecting a larger syntactic processing difficulty (Hagoort and Brown, 2000; Carreiras et al., 2004; Silva-Pereyra and Carreiras, 2007; Molinaro et al., 2008). Since so-called ‘semantic P600s’ (rather than N400s) have been observed for sentences containing thematic role reversals, it has also been suggested that P600s may reflect the resolution of mismatches between two or more distinct (e.g., semantic and syntactic) processing streams (Kuperberg et al., 2003; see also Hoeks et al., 2004; Kim and Osterhout, 2005). When semantic expectations are contradicted by the syntactic structure of the sentence, a processing cost is incurred (Kolk et al., 2003; van Herten et al., 2005). Yet others have suggested that processes underlying the P600 may comprise the ‘construction, revision, or updating of a mental representation of what is being communicated’ at multiple levels (Brouwer et al., 2012).

The finding of group differences both in latency and scalp topography of the P600 suggests the involvement of different processing routines for V-NP-object sentences in Attriters compared to native-controls. In Controls, this mild late effect reflects re-analysis and repair processes. When semantic cues that reliably support the respective theta roles are accessed early on (N400) and revision toward an object-relative interpretation is not costly, Controls elicited a smaller late P600 and arrived at higher acceptability ratings. Conversely, a stronger processing cost in Attriters seems to be related to their reduced use of semantic information and stronger expectations of number agreement between the sentence-initial noun and the subsequent verb. Relying on a ‘subject-first’ processing strategy typical for English, Attriters encountered a morphosyntactic number agreement violation on the verb, triggering the typical profile of a substantial P600 violation effect and leading to a low acceptability rating at the end of the sentence (suggesting they did not successfully reanalyze the structure). Consistent with this view, larger P600 amplitudes were associated with a higher degree of L2-English proficiency and a longer LoR. That increased L2 immersion and proficiency was associated with stronger morphosyntactic violation effects in response to V-NP-object sentences strongly suggests L2 influence on L1 morphosyntax and a shift in Attriters’ expectations during online sentence processing.

In fact, the latency and topographical differences in the P600 between Attriters and Controls are somewhat reminiscent of the patterns observed in the same exact participants while processing subject-verb number agreement violations during the same Italian experimental session (Kasparian et al., 2016). In response to number violations, Attriters elicited a large P600 effect beginning around 650 ms that was less posterior and shorter than in Controls. Their acceptability ratings for this outright agreement violation and the ‘apparent’ agreement violation in our present V-NP-object condition were also comparable. This similarity further supports our view that Attriters have a stronger subject-first preference than Controls, based on the influence of English word-order. This strong preference leads Attriters to diagnose a number mismatch between the verb and its preceding noun, even if the sentence is grammatically acceptable in Italian. Importantly, outright agreement violations in the Control group did *not* result in the P600 pattern we observe for our present V-NP-object condition. Their P600 for outright agreement violations was not delayed but started at the same time as the P600 in Attriters (i.e., around 650 ms). Correspondingly, outright violations were rated as less acceptable than the V-NP-object garden-path sentences.

A final point to discuss is whether the smaller and later P600 in Controls might be directly linked to the presence of an N400 due to component overlap (see Osterhout and Mobley, 1995; Osterhout, 1997; Steinhauer and Drury, 2012; Tanner et al., 2013, 2014; Tanner and Van Hell, 2014; Tanner, 2015). This possibility is compatible with our finding that the early portion of the N400 has a typical broad distribution with a centro-parietal maximum, whereas the later negativity (after 500 ms) is more frontal (see **Figure 4**). This is exactly the pattern one would expect if the late portion of the N400 and the early portion of a clearly more posterior P600 (significant after 850 ms) superimposed one another and canceled each other out. If component overlap is indeed the main reason for the absence of an earlier P600 (present only in Attriters), this would imply that *both* the P600 and the N400 observed in the Control group were underestimated, i.e., the actual N400 must have been even larger and must have lasted longer. If so, and given that the Attriters did not show any evidence of an N400 at all, this would illustrate just how different the processing strategies between the groups were. On the other hand, if the observed pattern was not influenced by component overlap, the finding of a substantially delayed P600 (starting around 850 ms) in the monolingual native speakers of the Control group is difficult to reconcile with the expected ERP profile for a number violation in this group (see previous paragraph) and would, again, point to distinct processing strategies compared to the Attriters.

### Processing of NP-V Sentences

The first contrast we discussed above compared the processing of the generally highly preferred V-NP-subject relative clause to a relatively difficult V-NP-object garden-path sentence. We saw that the monolingual Control group processed the latter like a garden-path and used semantic cues, whereas Attriters with strong exposure to English processed it like an outright morphosyntactic violation, as would be expected for English speakers. The second comparison of NP-V structures differs from this first comparison in various respects. First, when the disambiguating element is reached (verb), both noun phrases have already been encountered. Thus, the Control group that was shown above to use semantic cues can be expected to compute even stronger expectations based on the preliminary assignment of Actor and Undergoer to the available NPs (Bornkessel-Schlesewsky and Schlesewsky, 2013).

Second, whereas for Attriters (who employ a parsing strategy influenced by English), the present contrast is somewhat similar to the previous V-NP contrast, this may not be the case for the Controls. From the Attriters’ perspective, we again compare one sentence structure that is grammatical in English (V-NP-object) to a second structure that is ungrammatical in English (V-NP-subject). If Attriters are indeed generally influenced by English parsing preferences, we would expect another instance of morphosyntactic violation effects. By contrast, from the Controls’ perspective, we compare two structures that can both be described as garden path sentences. Whereas NP-V-object sentences are the preferred structure to express object relative clauses in Italian, they nevertheless constitute a non-preferred structure compared to the V-NP subject relative clauses discussed in the previous section, and should encourage Italian Controls to use semantic cues to disambiguate the structure.

The NP-V-object structure requires an Object-Subject-Verb analysis (e.g*., The thieves that the policeman arrests …*) and is (similar to English) a quite frequent construction in Italian. The NP-V-subject structure requires a Subject-Object-Verb analysis (e.g*., The policeman that the thieves arrests …*), which is not very frequent in Italian but has the potential advantage that the first NP (i.e., the referent of the relative pronoun) still serves as the subject of the relative clause (similar to the most preferred V-NP-subject structure). Nevertheless, in line with Di Domenico and Di Matteo’s (2009) behavioral study, these sentences received the lowest acceptability ratings in both groups (but were comparable to those for outright violations only in the Attriters).

In response to NP-V-subject garden path sentences in Italian, Controls elicited a small, late, posterior P600 starting around 900 ms. In Attriters, however, NP-V-s sentences elicited a strong, widespread N400-like negativity, followed by a larger frontal positivity and a more robust early P600 starting around 650 ms. As a whole, this pattern is (again) compatible with the assumption that the Control group processed the difference between conditions as a garden path, whereas the Attriters processed it like an outright violation. Given that Controls’ acceptability ratings displayed substantial differences between the two conditions, the rather weak ERP differences may be somewhat surprising. However, the notion that both sentences should be viewed as garden path structures may provide some explanation. As mentioned above, in both NP-[NP-V] constructions, both subject and object NPs had already been encountered before the disambiguating verb was presented, and our materials always provided reliable semantic cues as to which NP was a plausible Actor/Agent or a plausible Undergoer/Patient. According to both the Competition Model (MacWhinney and Bates, 1989) and the eADM model (Bornkessel-Schlesewsky and Schlesewsky, 2006/2008), Italian Control subjects were expected to use these semantic cues, either to predict the theta role assignment or to support the final analysis once the verb information became available. We assume that these processes were largely the same in *both* conditions, even though the less frequent NP-V-subject condition was identified as somewhat more difficult (eliciting a small P600) and resulted in a lower rating.

In Attriters, we find substantial ERP differences between the two conditions, consisting of a negativity followed by positivities. Interestingly, while the NP-V-object condition (which is grammatical in English) did not differ from the corresponding ERPs in the Control group, the NP-V-subject condition (which is ungrammatical in English) did (**Figure 9**).

The timing and distribution of the negativity suggest a mix between a LAN and an N400, with no hemispheric differences reaching statistical significance. In the literature, both LANs and N400s have been reported for number agreement violations, and both have been linked to lexical mismatch effects based on context-based morphological predictions (Molinaro et al., 2011; Tanner and Van Hell, 2014), or to lexical retrieval problems (Brouwer et al., 2012). An N400 would also be consistent with the view that computing the thematic roles requires access to lexical-semantic information (Deutsch and Bentin, 2001; Barber et al., 2004; Molinaro et al., 2008, 2011), if we assume that, at this point in the sentence, readers must determine which of the two presented nouns is the subject of the relative clause verb. In other words, even though Attriters do not seem to have used semantic cues as soon as they were provided by the NPs, once all NPs and the verb were available, they had to assign theta roles.

The presence/absence of the negativity cannot be reduced to component overlap (Osterhout and Mobley, 1995; Osterhout, 1997; Tanner et al., 2013, 2014; Tanner and Van Hell, 2014; Tanner, 2015), as the first (frontal) positivity was *larger* in the Attriters, i.e., in the group that also elicited the preceding negativity. Rather, Attriters and Controls seem to be engaging in different processing routines. We interpret the larger frontal positivity in Attriters as a P3a component that has been associated with a surprise effect and shift in attention (Squires et al., 1975; Polich, 2007). Similar frontal positivities were previously observed in temporary subject/object ambiguities in Italian *wh* constructions for target words disambiguating the more difficult object reading (Penolazzi et al., 2005). In our study, this frontal P3a was immediately followed by a large and early P600 that is indicative of a violation effect and corresponding processing costs, similar to the V-NP contrasts discussed above.

To summarize, Attriters who, due to English influence, were hypothesized to be less sensitive to semantic/thematic cues than to word-order preferences, elicited strong ERP violation effects on the verb in both V-NP-object and NP-V-subject constructions. For the same sentences, matched monolingual Italian control subjects demonstrated weaker ERP effects that are expected for native speakers processing these types of garden-path sentences. Since the two sentence conditions are ungrammatical in English, but grammatical in Italian, the group differences strongly point to distinct processing strategies. As predicted, Attriters seemed to have adopted parsing strategies from their predominantly used English L2.

### Implications for First Language Attrition

The present study investigated L1 attriters’ processing of a complex aspect of morphosyntax where the L1 and L2 either converge or diverge. Interestingly, our experimental design allowed us to examine neuroplasticity in Attriters’ processing routines for grammatical sentences in their L1, which happened to be ungrammatical in their L2. Similar to a few previous ERP studies (e.g., Thierry and Wu, 2007; Kasparian et al., 2010), this approach focuses on the influence of a seemingly ‘irrelevant’ language (English) on the presented language under investigation (Italian) and thus differs from testing morphosyntactic violations in the language presented, as is traditionally done in ERP language research. Moreover, in contrast to the few other studies using this approach to test the impact of L1 on L2, here we investigated the impact of L2 on L1. Our findings provide evidence of cross-linguistic influence from the L2 due to immersion and reduced L1 use, resulting not just in quantitative but also in qualitative changes in adult Attriters’ processing patterns, contrary to the findings reported in the only other published ERP research of L1 attrition investigating a different population of attriters (Bergmann et al., 2015). The present results further support and extend those reported in our Italian number agreement study (Kasparian et al., 2016), suggesting that more complex morphosyntactic manipulations result in greater processing differences between attriters and non-attriting native-speakers. In contrast to our other study, the present sentence structures were specifically selected to maximize differences in the cues that readers could rely on in Italian vs. English (i.e., semantic cues and word-order, respectively). Although behavioral studies have shown L2 to L1 transfer to occur in instances where a grammatical L2 feature is transferred to the L1 despite its ungrammaticality in L1 (e.g., Rippert and Kuiken, 2009), to our knowledge, this is the first demonstration where the opposite is true; namely, that high proficiency in a second language acquired in adulthood may render a *grammatical* sentence in one’s native language *ungrammatical* when processed in real-time.

Our ERP findings are in line with reports from eye-tracking studies of immersed L2-English speakers’ processing of L1 Spanish relative clauses (Dussias and Sagarra, 2007) where differences were found in relative clause attachment preferences in bilinguals with extensive L2-immersion, compared to native-Spanish monolingual speakers and bilinguals with limited L2 exposure. Although the bilinguals in Dussias and Sagarra’s study were not attriters (with limited/no exposure to the L1 in the L2 environment), their eye-tracking results parallel our ERP findings of changes in L1 processing as a result of extensive L2 exposure.

Although we are only at the very beginning of a long way to better understand the neurocognitive changes involved in first language attrition, the present results are very promising. In our opinion, they cannot be explained in terms of a mere “bilingualism effect” (i.e., a by-product of having compared monolingual Controls to bilingual Attriters), as even within Attriters, ERP responses are modulated by factors such as exposure, proficiency and LoR. These factors have been shown to modulate ERP response patterns in these same Attriters on other lexical-semantic and morphosyntactic properties, both in their L1 (Kasparian and Steinhauer, 2016; Kasparian et al., 2016) as well as in their L2 (Kasparian et al., unpublished). Interestingly, in the latter study, we showed reduced L1 activation (increased inhibition) during L2 processing in Attriters with less frequent L1 exposure/use and a longer LoR. These findings fit with frameworks of relative frequency of use and activation thresholds, where the more dominant language is associated with a higher baseline activation level and a better efficiency in inhibiting cross-linguistic competition (e.g., McDonald, 1987; MacWhinney, 1992; Kroll and Stewart, 1994; Jared and Kroll, 2001; Dijkstra and Van Heuven, 2002; Gollan et al., 2008).

In attrition research, the relationship between attrition effects observed in behavior and at the brain level is still largely unexplored. On the behavioral proficiency tasks we administered, Attriters scored numerically lower than Controls but did not differ significantly on any of the measures. However, their end-of-sentence acceptability judgments largely reflected the preferences observed during real-time sentence processing, namely that the two word-orders that are ungrammatical in English were judged as unacceptable. This fits with the argument made by Steinhauer et al. (2009) that structure-specific proficiency (rather than overall proficiency) best predicts ERP response patterns. However, this was also an interesting finding, given that in our number agreement study (Kasparian et al., 2016), we found group differences in ERP responses but not in acceptability ratings (see McLaughlin et al., 2004 for a similar finding in L2 vocabulary acquisition). The nature of the sentences may explain this discrepancy; in our number agreement experiment, we manipulated the agreement between three sentence constituents (subject, verb, and modifier), giving rise to different combinations of (dis)agreement that may have resulted in a less straightforward acceptability judgment task than in our present study. In addition, our current design directly tested a morphosyntactic area where the two languages either converge or clash. It is likely that the language areas and tasks on which Attriters differ most from native-Controls are those which tap directly into the effects of L2 influence on L1.

In sum, the present study provides evidence of neurocognitive change due to language learning in adulthood. Our results revealed both quantitative and qualitative changes in L1 morphosyntactic processing patterns of Italian native-speakers who had lived in an exclusively monolingual L1 context until adulthood. Thus, even an “entrenched” L1 grammar is subject to change after a prolonged period of L2 immersion and reduced L1 exposure/use. As the L2 takes the lead, its acquisition and use induces changes to attriters’ L1 neurocognitive processes and results in differences from non-attriting native-speakers. In the present study, we have shown that a key factor in promoting these changes in attriters is the influence of the L2 on the L1, both in terms of the language pairing and related cross-linguistic differences, as well as in terms of increasing amount of L2 exposure/use and proficiency relative to the L1.

## Ethics Statement

This study was carried out in accordance with the recommendations of the McGill University Faculty of Medicine Institutional Review Board and the Ethical Committee for Human Research, University of Trento. All subjects gave written informed consent in accordance with the Declaration of Helsinki. The protocol was approved by the Institutional Review Board (#A06-B30-11A) and the Ethical Committee for Human Research (#2013-003) of the respective institutions.

## Author Contributions

KK and KS contributed equally to the experimental design of the study. KK created the experimental stimuli, programmed most parts of the experiment, recruited and tested participants in Italy and in Canada (with the help of research assistants) and conducted a large part of the data analyses. KS contributed to programming and data analyses and oversaw the project. KS and KK contributed equally to data interpretation. The manuscript was written by KK with input from KS.

## Conflict of Interest Statement

The authors declare that the research was conducted in the absence of any commercial or financial relationships that could be construed as a potential conflict of interest.

## Footnotes

1 Acceptability means per condition were not reported in the paper but were obtained in a personal communication with the authors. The order of acceptability of the sentence conditions was #1, 4, 2, 3.

2 It is not within the scope of the present study to disentangle between these views.

3 Participants unanimously considered their AoA of English to coincide with their age of immersion into English (i.e., immigration), given that their exposure to English within the Italian school system was only minimal.

4 Our rationale for this decision was based on several reasons. First, the word-orders differed between the first pair of conditions (V-NP) and the second pair of conditions (NP-V), resulting in differences in the sentence context that appeared prior to the relative clause verb (where we time-locked our analyses). As reported, we also used a different baseline correction for the NP-V analysis. Similarly, representative time windows to best capture the relevant ERP effects differed between the two word-orders and would have required to introduce additional time windows. Including ‘word-order’ (V-NP; NP-V) as a factor with relative clause type (subject, object) and group (attriters, controls) along with time-window and topographical factors would have resulted in very complex ANOVA that would most likely result in many significant interactions that would distract from the actual patterns rather than clarify them. For the sake of clarity, we believe reporting the two types of violations in separate sections is the only feasible way.

5 The new baseline worked against our hypotheses, as the original baseline overestimated the early negativity found in Attriters (but not in Controls). Plots with the original baseline correction are provided in Supplementary Materials.

6 We analyzed the results separately for VN orders (2) and NV orders (2) because participants were categorized as high- or low-raters based on their acceptability judgments on the 2 unpreferred conditions (i.e., V-NP-object and NP-V-subject), and it was not always the case that the same participants were “low” raters for both unpreferred orders.

7 Attriters were not slower across the board than Controls for all experiments conducted as part of the larger study. However, they also exhibited slower response times in a study of Italian number agreement processing (Kasparian et al., 2016) which tested similarly complex sentences.

8 Despite large differences between high and low rater Controls in terms of N400 amplitudes, *F*-values, and *p*-values, the “*Condition* × *Rater*” interaction did not reach significance (*p* = 0.1). Sample size differences may have played a role. However, an important point to emphasize is that we would expect the opposite if the N400 were an indicator of a violation effect.

9 The difference between high rater Controls and high rater Attriters was marginal [*F*(1,25) = 3.04, *p* = 0.09].

10 The *C* × *AP* interaction in the global ANOVA (in absence of a significant *C* × *AP* × *G* interaction) may be interpreted as an indication that even the Controls may also have some kind of a significant P600, which, however, was restricted to PZ. Alternatively, the presence of a *C* × *AP* interaction in the Controls can also be due to a relative frontal negativity, i.e., what’s shared across groups is simply the gradient of ‘more positive’ potentials at more posterior electrodes (but in absence of a true P600). The latter pattern was what we found in a follow-up analysis at Pz. A marginal *C* × *G* interaction was found at Pz [*F*(1,51) = 3.15, *p* = 0.08], further supporting a more robust P600 effect and a more laborious revision in Attriters [*F*(1,22) = 11.75, *p* < 0.005] than Controls (*p* > 0.1).

11 Readers familiar with the recent debate on ‘semantic P600s’ and ‘semantic illusions’ may notice that our N400 findings (as well as those in Mecklinger et al., 1995) are problematic for most accounts proposed to explain the processing of role reversals (e.g., Brouwer et al., 2012, and other models discussed in their paper). We mention this debate only briefly, as a broader discussion is beyond the scope of our paper. See Bourguignon et al. (2012) and Bornkessel-Schlesewsky and Schlesewsky (2013) for more N400 findings in role reversals that are difficult to explain by semantic priming alone.

12 To better assess the reliance on semantic cues for sentence interpretation, it would have been informative to test a fully balanced set of stimuli using both reversible (i.e., semantically neutral) and non-reversible (i.e., semantically biasing) sentences (see Mecklinger et al., 1995). However, given that the present study was one out of four experiments embedded into the same Italian testing session, it was not possible to present a full set of 16 conditions (8 main conditions counterbalanced in singular/plural number). For a fully balanced design including reversible sentences we hypothesize that a semantic facilitation effect would be found in Controls but not Attriters.
