# Supplementary material for: When the Second Language Takes the Lead: Neurocognitive Processing Changes in the First Language of Adult Attriters
Source: Front Psychol. 2017 Mar 30;8:389. doi: 10.3389/fpsyg.2017.00389 (PMC5371681; doi:10.3389/fpsyg.2017.00389)
Supplement: Supplementary file 1 [file Data_Sheet_1.pdf]

Supplementary Material

When the Second Language Takes the Lead: Neurocognitive Processing Changes in the First Language of Adult Attriters

Kristina Kasparian*, Karsten Steinhauer* **(McGill University)**

*** Corresponding Authors:** kristina.kasparian@mail.mcgill.ca, karsten.steinhauer@mcgill.ca

1 Supplementary Data: Baseline settings for NP-V contrasts

As reported in our main article, the baseline interval used for our published analysis of the NP-V contrasts was 0 to 1200 ms and thus comprised almost the entire average window rather than a standard pre-stimulus interval of 100 or 200 ms. Since different baseline settings can have a dramatic impact on the resulting ERP effects (Steinhauer & Drury, 2012), using such an atypical baseline interval must be justified. The following sections provide this justification.

1.1 Standard baseline: -200 to 0 ms

In line with our V-NP analyses, our first attempt to analyze the NP-V contrasts was also done using a standard pre-stimulus baseline interval from -200 to 0 ms. The corresponding results for both Controls and Attriters are illustrated below in **Figure SM_1**. What we found in the Control group was a (non-significant) small frontal positivity around 500 ms and a (significant) subsequent small posterior P600 (roughly between 600 and 800 ms). At central electrodes, most peaks across the entire time window were more positive in the violation condition (see black circles), potentially pointing to a mild shift of that waveform towards positive amplitudes due to a weak baseline artefact. In Attriters, however, we observed an implausible ERP pattern that was dominated (at least at frontal and central sites) by an extremely early negativity (starting at 0 ms) and a similarly distributed late negativity after 1000 ms, suggesting significant baseline problems (see black arrows in Fig. SM_1B). In addition, we found a significant fronto-central positivity and a significant parietal P600.

As discussed in detail by Steinhauer and Drury (2012), ERP differences starting at 0 ms or – as in our present case – even a little earlier, are physiologically implausible and cannot be real effects unless they are due to differences triggered by previous words further upstream. Quite often, these early differences are artifacts due to problems during baseline correction. In the latter case, standard baseline correction 'over-corrects' a *real* pre-stimulus difference by shifting the entire waveform of one condition towards the positive or negative amplitude range. If so, the duration of such artefactual differences between conditions is in principle unlimited (or may be limited by certain filter settings). This is exactly the scenario that we see in **Figure SM_1B** for the Attriters: the early negativity (starting at 0 ms) seems to re-occur

after 1000 ms and disappears only between approximately 600 and 1000 ms, i.e., in a time window where it is plausibly cancelled out by a real P600 effect. (This pattern is somewhat reminiscent of sustained frontal negativities superimposed by a P600, as discussed by Steinhauer and Drury, 2012.) In other words, the standard pre-stimulus baseline was an inappropriate choice for the current NP-V contrast, at least in the group of attriters.

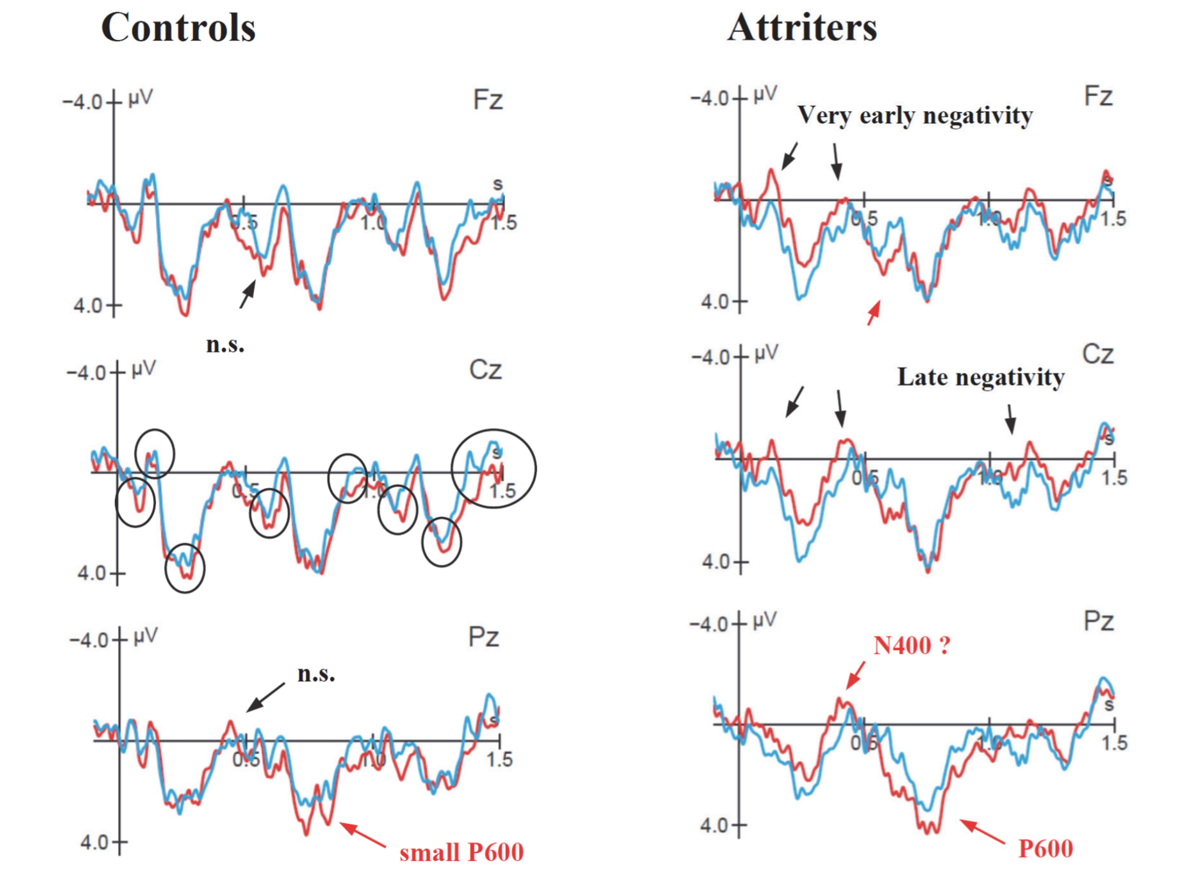

(A) **(B)**

Figure SM_1: NP-V effects with standard baseline (-200 to 0 ms), displayed at midline electrodes, **(A)** in Italian monolingual Controls (left panel) and **(B)** in Attriters (right panel). As in Figures 7 and 8 of the main paper, the red line represents the more difficult **NP-V-s** condition that is ungrammatical in English.

In order to better understand our data, we ran additional analyses further upstream (e.g., at the onset of the sentence or time-locked to the complementizer *"che"*) and could verify condition differences triggered by previous words that contaminated the standard baseline interval in the Attriters (not illustrated). The next step was to identify an alternative, valid baseline interval. Possible options include (i) extended pre-stimulus baselines (e.g., -1000 to 0 ms), (ii) distant baselines (e.g., -1000 to -800 ms), (iii) post-stimulus onset baselines (e.g., 0-200 ms), and (iv) a baseline interval comprising the entire average window (e.g., -200 to 1500 ms), all of which have certain advantages and disadvantages. Since the early negativity in Figure SM_1B increases in amplitude between 100 and 500 ms (especially at CZ and PZ, but also at other electrodes), there is a likelihood that only parts of its amplitude are due to an artifact, while other parts may be real effects (e.g., an N400). That is, certain baselines (especially long post-

stimulus onset baselines) may over-estimate and over-correct the artifact. After applying various different baseline intervals, we decided that the 0 to 1200 ms interval was the most appropriate one, which reduced the impact of pre-stimulus artifacts while at the same time avoiding over-corrections of potentially real negativities in the Attriters' ERP.

1.2 Our second approach – a baseline from 0 to 1200 ms

The ERP patterns resulting from the adjusted 0-1200 ms baseline are illustrated in **Figure SM_2**. Using this alternative baseline instead of the standard baseline did not really change the ERP pattern (i.e., the differences between conditions) of the **Control group** (Fig. SM_2A). Note however, that overall the ERP waveforms of both conditions have been shifted more towards the negative amplitude (this holds true for the Attriters as well). Even though the ERPs look slightly different, the only significant ERP difference between conditions is, again, the small posterior P600 between roughly 600 and 800 ms (see arrows). Instead of the non-significant small frontal positivity in Figure SM_1A, we now see a small central negativity similar to that at PZ (black arrows) which, however, also fails to reach significance. Overall, the data look more convincing than those in Figure SM_1, because the ERPs of both conditions are almost indistinguishable during the first 400 ms or so as well as at the end of the window (including at central sites, where the standard baseline had resulted in differences).

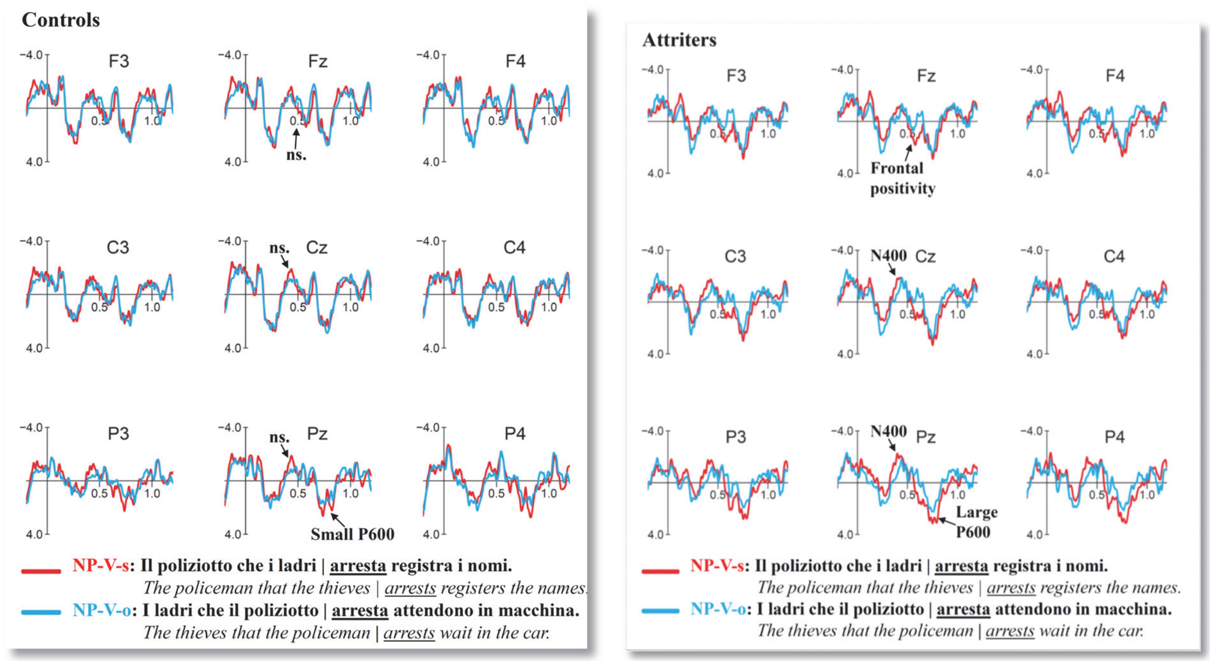

(A) **(B)**

Figure SM_2: NP-V effects with adjusted baseline (0 to 1200 ms), **(A)** in Italian monolingual Controls (left panel) and **(B)** Attriters (right panel). Note that these plots correspond to Figures 7 and 8 of the main paper and are repeated here for convenience.

In contrast, the ERPs for the **Attriters** are significantly affected by the new baseline. As expected, the early negativity at frontal and central sites is largely gone, only a relatively small (but significant) negative difference between 300 and 500 ms remains at central and parietal

electrodes (N400). In addition, we see both a large frontal positivity around 600 ms (P3a) and a large parietal P600 between 600 and 1000 ms. As with the Control group, overall the Attriters' ERPs computed with this baseline look more convincing than those in Figure SM_1.

Importantly, these adjusted analyses also display the differences elicited by previous words that caused the problems in our initial analysis using the standard baseline. That is, frontal and central sites display a considerable difference prior to target word onset, i.e., in the standard baseline window (-200 to 0 ms).
